# Triple-negative breast cancer in Peru: 2000 patients and 15 years of experience

**DOI:** 10.1371/journal.pone.0237811

**Published:** 2020-08-24

**Authors:** Gabriel De-la-Cruz-Ku, Marianne Luyo, Zaida Morante, Daniel Enriquez, Mecker G. Möller, Diego Chambergo-Michilot, Lucero Flores, Renato Luque, Antonella Saavedra, Miguel E. Eyzaguirre-Sandoval, María G. Luján-Peche, Naysha Noel, Hafid Calderon, Cesar Razuri, Hugo A. Fuentes, Jose Manuel Cotrina, Silvia P. Neciosup, Jhajaira Araujo, Alexandra Lema, Joseph Pinto, Henry L. Gomez, Bryan Valcarcel

**Affiliations:** 1 School of Medicine, Universidad Científica del Sur (UCSUR), Lima, Peru; 2 Universidad Peruana Cayetano Heredia, Lima, Peru; 3 Instituto Nacional de Enfermedades Neoplasicas, Lima, Peru; 4 Division of Surgical Oncology, University of Miami Miller School of Medicine, Jackson Memorial Hospital/Sylvester Comprehensive Cancer Center, Miami, FL, United States of America; 5 Tau-RELAPED Group, Trujillo, Peru; 6 Sociedad Científica de Estudiantes de Medicina Humana (SCIEM UCSUR), Lima, Peru; 7 Hospital Nacional Dos de Mayo, Lima, Peru; 8 Unidad de Investigación Básica y Translacional, Oncosalud-AUNA, Lima, Peru; 9 Health Sciences Faculty, Universidad de Cuenca, Cuenca, Ecuador; 10 Milken Institute School of Public Health, The George Washington University, Washington, DC, United States of America; Chang Gung Memorial Hospital at Linkou, TAIWAN

## Abstract

**Background:**

Epidemiological studies commonly identify the clinical characteristics and survival outcomes of patients with breast cancer at five years. Our study aims to describe the sociodemographic, clinicopathological characteristics and determine the long-term event-free survival (EFS) and overall survival (OS) of a Peruvian population with triple-negative breast cancer.

**Methods:**

We reviewed the medical records of new cases treated at a single institution in the period 2000–2014. The survival analysis included patients with stages I-IV. Survival estimates at 10 years were calculated with the Kaplan-Meier method and compared with the Log-rank test. We further used multivariate Cox regression analysis to calculate prognostic factors of recurrence and mortality.

**Results:**

Among the 2007 patients included, the median age at diagnosis was 49 years (19–95 years). Most patients presented histologic grade III (68.7%), tumor stage II (34.2%), and III (51.0%) at diagnosis. Local and distant relapse was present in 31.9 and 51.4% of the patients, respectively. The most frequent sites of metastasis were the lungs (14.5%), followed by bone (9.7%), brain (9.6%), and liver (7.9%). The median follow-up was 153 months. At 3, 5, and 10 years, the EFS of the population was 55%, 49%, and 41%, respectively, while the OS was 64%, 56%, and 47%, respectively. Moreover, an N3 lymph node status was the most important prognostic factor for both disease relapse (HR: 2.54, 95% CI: 2.05–3.15) and mortality (HR: 2.51, 95% CI: 2.01–3.14) at ten years. An older age and higher T staging were associated with a worse OS, while patients who received radiotherapy and adjuvant chemotherapy had better survival rates.

**Conclusion:**

The sociodemographic features of Peruvian patients with TNBC are similar to those of other populations. However, our population was diagnosed at more advanced clinical stages, and thus, EFS and OS were lower than international reports while prognostic factors were similar to previous studies.

## Introduction

Breast cancer remains the most prevalent and incident cancer in most countries. The World Health Organization estimated 2.1 million new cases of female breast cancer in 2018 [1]. The triple-negative breast cancer (TNBC) subtype, which is negative for the progesterone receptor (PR), estrogen receptor (ER), and human epidermal growth factor 2 receptor expression (HER2), accounts for approximately 10% of all the breast cancers [2]. However, a previous study identified a prevalence of 21.3% in a single Peruvian center [3].

The TNBC subtype is more aggressive and has worse survival outcomes compared to non-TNBC [4]. Patients with TNBC are more likely to be younger, obese, have a family history of breast cancer and non-Hispanic black women [5–7]. Moreover, Latin American population have a greater percentage of TNBC diagnosis, as well as HER2+ [8, 9]. Nevertheless, studies have not established if these demographic characteristics have a real impact on survival prognosis. In contrast, pathologic tumor features (large tumor size, lymph node involvement, and advanced stages) have a clear association with worse survival outcomes [10, 11].

Nonetheless, few reports have addressed the long-term survival outcomes of women with TNBC [11–13]. Haque *et al*. [12] reported an overall survival (OS) of 66% in TNBC patients at 20 years, while Li *et al*. [13] described 80% at seven years. Furthermore, Chandra *et al*. [11] reported an OS of 75% at eight years with a median follow-up of 42 months.

On the other hand, social environment has been described as having a negative impact on patient survival in Peru and other countries. This is due to insufficient access to health care services and unawareness of diagnostic tests [14]. There are scarce data on the clinicopathological features of TNBC in South America. Thus, the purpose of our study was to identify the sociodemographic, clinicopathological and therapeutic characteristics, as well as patient outcomes including long-term event-free survival (EFS) and OS in Peruvian women with TNBC. In addition, we report disease recurrence and prognostic factors of mortality at 10 years.

## Materials and methods

### Patients and design

This was a retrospective cohort study, approved by the institutional review board of the National Institute of Health Disease (INEN, “Instituto Nacional de Enfermedades Neoplásicas”). We included incident TNBC patients treated at the INEN from 2000 to 2014. The medical records were accessed from January 2016 to January 2017 and the follow-up data was updated in February 2020. Cases which were lost to follow-up after surgery were excluded from the study. Patients were treated with surgical or medical therapy. Patients undergoing surgery received breast-conserving surgery (BCS) plus radiotherapy, chemotherapy, or both; and total mastectomy (TM) [15] plus chemotherapy or radiotherapy. Medical treatment consisted of chemotherapy. Patients with positive margins underwent a re-excision procedure. Regardless of the treatment (surgical or medical), all cases were included. Some patients did not received chemotherapy for the following reasons: will of the patients or decision for palliative care due to metastatic disease. Patients were identified by immunohistochemical confirmation of TNBC (estrogen-receptor [ER] negative or <1%, progesterone-receptor [PR] negative or <1%, and human epidermal growth factor receptor 2 [HER2] negative or <1%), tumors with a HER2 inconclusive staining intensity were evaluated with fluorescence in situ hybridization (FISH), for confirming the negativity with a result less than 2. In regard to molecular and genetic tests as Oncotype and BRCA-1/2, the National Health Insurance do not provide these tests for the population, with an exception of FISH for HER2 expression, which could be done in the case of inconclusive immunohistochemical test. Some patients could be tested in other private institutions; however, those results were not registered. The 8^th^ edition of the American Joint Committee on Cancer (AJCC) was employed to classify the tumor stage of the patients. Moreover, the body mass index (BMI) was classified as underweight (<18.50 km/mt^2^), normal (18.50–24.99 km/mt^2^), overweight (25.0–29.99 km/mt^2^), obese I (30.0–34.99 km/mt^2^), and obese II (≥35 km/mt^2^). Moreover, we described the region of birth of patients as follows: Lima (capital of Peru), Coast (excluding Lima), Mountains and Jungle.

### Follow-up

The study follow-up started at the time of first treatment; therefore, we defined OS until the date of an event (death or end of the study), and EFS until locoregional or distant relapse, progression, death, or end of the study. We ensured a minimum observation of 36 months for the OS unless death occurred first. Breast cancer recurrence was identified by computed tomography or magnetic resonance and a biopsy.

### Statistical analysis

We used the median and range to report the age and BMI of the patients. Furthermore, the Chi-square test examined the differences between qualitative variables, and the McNewar test was used for quantitative variables. Moreover, the Kaplan-Meier method was used to compute the survival curves, and the Log-rank test estimated the differences between tumor stages. Furthermore, the prognostic factors for disease recurrence and mortality were estimated with multivariate Cox regression analysis and reported with proportional hazard ratios [16] and a 95% confidence interval [9]. We excluded cases with missing values of the variables menopausal status and histologic grade, which yielded a model adjusted for the following variables: age, breast or ovarian family history, menopausal status, T staging, N staging, histologic grade, chemotherapy and radiotherapy. The results were considered statistically significant with a p value < 0.05. Both multivariate models were assessed with the command “cox.zph” and assumed the proportional HR assumption. Data were analyzed using the software R 3.5.3 version and the packages “survival” and “survminer”.

### Ethical aspects

This study was approved by the Institutional Research Ethics Committee of the “Instituto Nacional de Enfermedades Neoplásicas”. We blinded the identity of the participants, replacing their information with unrelated codes (e.g. 1, 2).

## Results

### Baseline demographic characteristics

**Fig 1:**
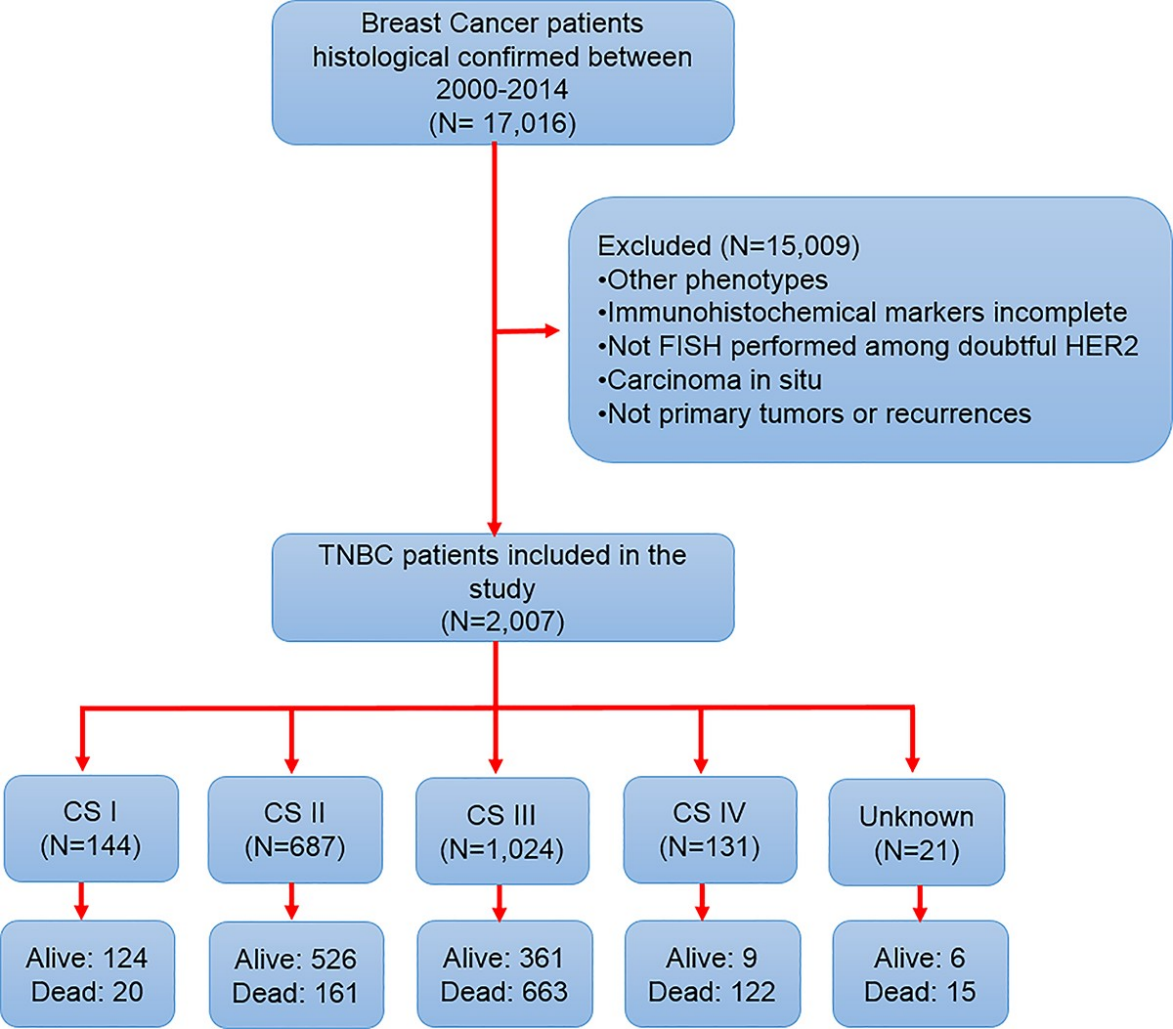
Graphic schematization of patient inclusion in the study.

We analyzed a total of 2007 patients diagnosed with TNBC (Fig 1). Table 1 shows the baseline sociodemographic characteristics of the overall population. The median age at diagnosis was 49 years (19–95 years), and most patients were between 40–59 years of age. We found a high prevalence of overweight (34.7%), postmenopausal status (54.6%), and family history of breast or ovarian cancer in a first- or second-degree relative (10.4%). Furthermore, our study showed that hypertension and diabetes mellitus (DM) had a prevalence of 9.9% and 8.1%, respectively. In fact, hypertension was more common in the overweight (8.8%) and obese patients (19.7%). Similar results were obtained for DM, with 8.2% and 14.0% in overweight and obese patients, respectively. Moreover, our patients were more likely to have T2 or T4 T staging, N0-1 N staging, AJCC tumor staging II-III, and histologic grade III (Table 2).

**Table 1:** Baseline sociodemographic characteristics of the overall population.

Characteristics	Total population (N = 2,007)	Percentage (%)/range
Age (median)	49	19–95
Age (years)		
<30	64	3.2
30–39	373	18.6
40–49	602	30.0
50–59	512	25.5
60–69	298	14.8
≥70	158	7.9
Region of birth		
Lima	579	28.85
Coast (excluding Lima)	811	40.41
Mountains	479	23.87
Jungle	138	6.87
Body mass index (median)	26.84	14.67–54.42
Body mass index		
Underweight	22	1.1
Normal	553	27.6
Overweight	696	34.7
Obese I	335	16.7
Obese II	93	4.6
Obese III	23	1.1
Missing	285	14.2
Menopausal status		
Premenopause	889	44.3
Postmenopause	1095	54.6
Missing	23	1.1
Family history		
Cancer family history	581	28.9
Breast or ovarian cancer in 1^st^ degree relative	117	5.8
Breast or ovarian cancer in 2^nd^ degree relative	93	4.6
Breast or ovarian cancer in 3^rd^ degree relative	36	1.8
Breast or ovarian cancer in ≥2 relatives	58	2.9
Reproductive history		
Number of children (median)	2	0–14
Number of pregnancies (median)	3	0–22
Patients with history of abortion	740	36.9

**Table 2:** Baseline clinicopathological and therapeutic characteristics of the overall population.

Characteristics	Total population (N = 2,007)	Percentage (%)
T classification		
T0	5	0.2
T1	191	9.5
T2	669	33.3
T3	229	11.4
T4	798	39.8
Missing	115	5.7
N classification		
N0	651	32.4
N1	715	35.6
N2	302	15.0
N3	200	10.0
Missing	139	6.9
Tumor stage		
Stage I	144	7.2
Stage II	687	34.2
Stage III	1,024	51.0
Stage IV	131	6.5
Missing	21	1.0
Laterality		
Right	944	47.0
Left	1,027	51.2
Bilateral	36	1.8
Histologic grade		
Grade I	18	0.9
Grade II	305	15.2
Grade III	1,378	68.7
Missing	306	15.2
Histological Subtype		
Ductal	1,860	92.7
Lobular	46	2.3
Medullar	32	1.6
Metaplastic	16	0.8
Epidermoid	8	0.4
Apocrine	6	0.3
Mucinous	4	0.2
Secretor	2	0.1
Adenoid cystic	2	0.1
Others	31	1.5
Surgery as first line of treatment	1,064	53.0
Surgery	1,515	75.5
Conservative	488	32.2
Mastectomy	1,027	67.8
No Surgery	485	32.0
Neoadjuvant Chemotherapy	744	37.1
Adjuvant Chemotherapy	987	49.2
Radiotherapy	1,012	50.4
Local relapse		
Yes	677	33.7
No	1,330	66.3
Distant relapse		
Yes	1,047	52.2
No	960	47.8

### Surgical and treatment characteristics

Among the medical therapies administered, 37.1% of the patients received neoadjuvant chemotherapy, 50.8% received adjuvant chemotherapy and 50.4% adjuvant radiotherapy. Surgery was the first line of treatment in 53% of the population (Table 2). Of the 1515 patients who had surgery (75.5%), most underwent TM (72.5%). Patients undergoing BCS (32.2%) had a higher percentage of positive margins, and therefore a high frequency of re-excision. However, the proportion of positive vascular permeation and perineural invasion was higher in patients with TM (Table 3).

**Table 3:** Surgical characteristics of the population according to type of surgery.

Characteristics	Breast-conserving surgery (%) N = 488	Total mastectomy (%) N = 1,027	P-value
Margins status			<0.001
Negative	317 (64.9)	944 (91.9)	
Positive	137 (28.1)	36 (3.5)	
Missing	34 (7.0)	47 (4.6)	
Re-excision			<0.001
No	300 (61.5)	946 (92.1)	
Yes	168 (34.4)	34 (3.3)	
Missing	20 (4.1)	47 (4.6)	
Vascular permeation			0.004
Negative	182 (37.3)	438 (42.6)	
Positive	129 (26.4)	457 (44.5)	
Missing	177 (36.3)	132 (12.9)	
Perineural invasion			0.002
No	220 (45.1)	641 (62.4)	
Yes	47 (9.6)	238 (23.2)	
Missing	221 (45.3)	148 (14.4)	

### Treatment outcomes and survival analysis

**Fig 2:**
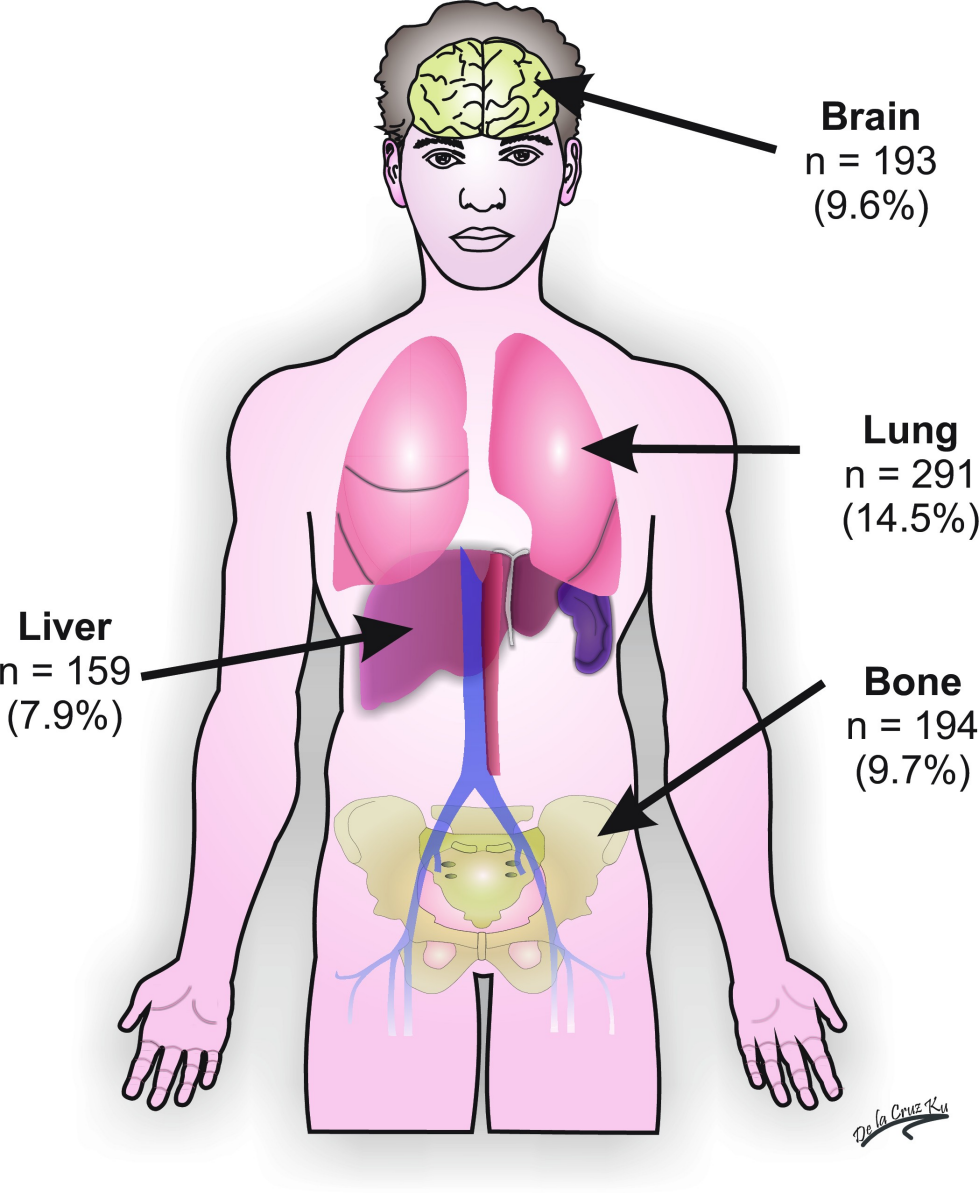
Frequency of site of metastasis in Peruvian triple-negative breast cancer patients.

**Fig 3:**
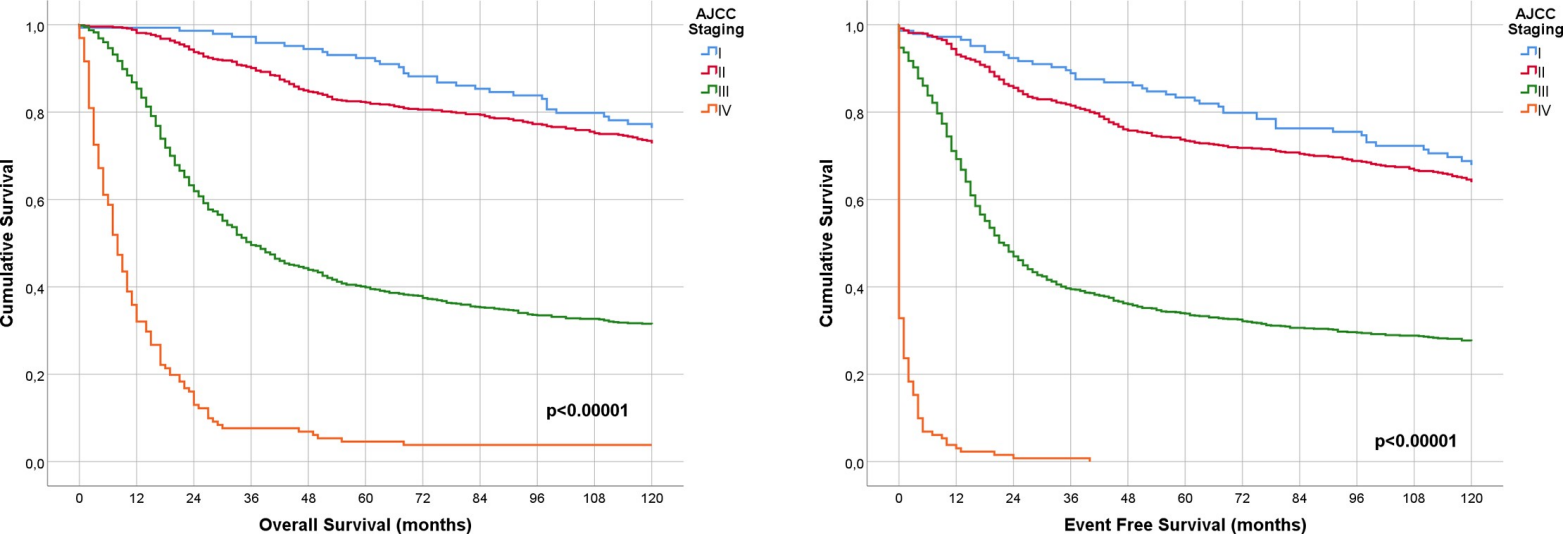
(A) Overall survival according to AJCC Staging. (B) Event-free survival according to AJCC Staging.

The median follow-up was 153 months (12.75 years), and after 10 years of observation, the rate of local recurrence was 31.9%, and 51.4% for distant recurrence. The most frequent sites for breast cancer metastasis were the lungs (14.5%), followed by bone (9.7%), brain (9.6%) and liver (7.9%) (Fig 2). Table 4 shows that the OS of the entire population at three, five, and ten years was 64%, 56%, and 47%, respectively. Moreover, the EFS and OS of patients continually decreased according to AJCC staging at three, five, and ten years (Table 4, Fig 3A and 3B). The prognostic factors for both mortality and disease recurrence were an older age, higher T or N status, and absence of chemotherapy and radiotherapy (Table 5).

**Table 4:** Overall survival and event-free survival probabilities of patients with tumor stage I-IV.

Survival outcomes	Time periods			P-value
	3y (%)	5y (%)	10y (%)	
Event-free survival				<0.00001
Total population	55	49	41	
Stage I	89	83	68	
Stage II	82	73	65	
Stage III	40	34	28	
Stage IV	1	0	0	
Overall survival				
Total population	64	56	47	
Stage I	97	92	76	<0.00001
Stage II	90	82	73	
Stage III	50	40	31	
Stage IV	8	5	4	

**Table 5:** Prognostic factors for overall survival and event-free survival of patients with tumor stage I-IV.

Characteristics	Event-free survival						Overall Survival					
	Analysis I			Analysis (a)			Analysis I			Analysis (a)		
	cHR	95% CI	p value	aHR	95% CI	p value	cHR	95% CI	p value	aHR	95% CI	p value
Age	1.01	1.001–1.01	**0.018**	1.01	1.003–1.013	**0.002**	1.01	1.004–1.01	**<0.0001**	1.01	1.005–1.015	**<0.001**
Family history of breast and/or ovarian cancer												
No	1.00			1.00			1.00			1.00		
Yes	0.87	0.73–1.02	0.09	1.01	0.84–1.23	0.89	0.84	0.71–1.00	0.053	0.98	0.80–1.20	0.87
T staging												
T1-2	1.00			1.00			1.00			1.00		
T3-4	2.59	2.30–2.91	**<0.0001**	1.21	1.15–1.27	**<0.001**	2.90	2.56–3.29	**<0.0001**	1.24	1.17–1.31	**<0.0001**
N staging												
N0	1.00			1.00			1.00			1.00		
N1	2.01	1.74–2.31	**<0.0001**	1.54	1.30–1.81	**<0.001**	1.97	1.70–2.29	**<0.0001**	1.41	1.19–1.67	**<0.001**
N2	2.55	2.15–3.02	**<0.0001**	1.92	1.58–2.34	**<0.001**	2.83	2.37–3.37	**<0.0001**	1.98	1.62–2.42	**<0.001**
N3	3.09	2.56–3.73	**<0.0001**	2.52	2.03–3.13	**<0.001**	3.34	2.84–4.05	**<0.0001**	2.49	1.99–3.11	**<0.001**
Histologic grade												
Grade I-II	1.00			1.00			1.00			1.00		
Grade III	1.03	0.88–1.19	0.73	0.98	0.84–1.14	0.78	1.08	0.93–1.27	0.33	0.97	0.84–1.14	0.74
Chemotherapy												
No	1.00			1.00			1.00			1.00		
Adjuvant	0.53	0.46–0.62	**<0.001**	0.69	0.57–0.84	**<0.001**	0.48	0.41–0.56	**<0.001**	0.64	0.52–0.78	**<0.001**
Neoadjuvant	1.38	1.20–1.59	**<0.001**	1.18	0.97–1.44	0.10	1.41	1.22–1.63	**<0.001**	1.24	1.02–1.51	**0.031**
Radiotherapy												
No	1.00			1.00			1.00			1.00		
Yes	0.87	0.78–0.97	**0.010**	0.87	0.76–0.99	**0.04**	0.84	0.95–0.94	**0.003**	0.84	0.72–0.96	**0.015**

HR: hazard ratio; c: crude analysis; a: adjusted analysis for age, breast and/or ovarian cancer family history, CI: confidence interval; T staging, N Staging, histological grade, chemotherapy and radiotherapy.

### Characteristics and outcomes of population who received neoadjuvant chemotherapy

There were 744 patients that received neoadjuvant chemotherapy (NAC). Most participants did not receive NAC were ≥50 years old (50.6% vs. 44.2%, p = 0.022) and were postmenopausal (57.8% vs. 50.7%, p = 0.002). Moreover, the majority of T3-4 (86% vs 33.9%, p<0.0001), N1+ (81.3% vs. 54.9%, p<0.0001), and AJCC Stage III (81% vs. 34%, p<0.001) had NAC. Higher histological grades were related to NAC (84.8% vs. 78.9%, p = 0.003). Most of the patients who received NAC were treated with radiotherapy (58.9% vs 45.5%, p<0.0001). However, local (42.3% vs 28.7%, p<0.001), distant recurrence (71.1% vs. 41.0%), and death (74.9% vs. 50.9%) were more frequent in patients that received NAC (S1 Table).

Regarding the prognostic factors in the population with NAC, we controlled the following variables in the model: age, family history of breast and/or ovarian cancer, T staging, N staging, histological grade, and radiotherapy. A higher T staging (T3-4 vs T1-2, HR: 1.77; 95% CI:1.29–2.44) and N staging (N2 vs N0; HR:1.68; 95% CI:1.24–2.29; N3 vs N0; HR: 2.70; 95%CI: 1.87–3.88) were associated with worse OS. On the other hand, radiotherapy was associated with better outcomes in OS (HR:0.69; 95% CI: 0.57–0.74) (S2 Table).

## Discussion

The present study provides the sociodemographic, clinicopathological and survival profile of TNBC patients over 15 years in Peru. Our results show that the patients studied were mostly middle-aged women, overweight or obese, postmenopausal, or had tumor stage III at diagnosis. Moreover, a higher tumor stage worsened the EFS (local or distant) and OS rates in contrast to patients with stages I-II. The predominant prognostic factors for mortality and disease relapse in the multivariate Cox regression analysis were advanced T and N status.

Our cohort shares similar demographic, clinical, and pathological characteristics to those described in previous reports. The age at diagnosis of patients with TNBC clusters towards younger females compared to other breast cancer subtypes. According to several studies, the median age at diagnosis does not exceed 50 years [5, 6], and as expected, more than half of our cohort was under this cutoff point. Moreover, studies have identified an association between TNBC and an increased BMI. For example, Trivers *et al*. [6] found that TNBC patients had an almost 2-fold greater likelihood of being obese. Likewise, Mowad *et al*. [17] evaluated 183 patients with TNBC and found that the majority were overweight (23%) or obese (64%). However, they did not find an association between poor prognosis and OS and disease-free survival (DFS), even though the patients with an increased BMI had larger tumor sizes, higher T stages, and tumor stages. Accordingly, we found that the most predominant was the overweight group (38.1%), and a significant number of patients were obese (almost 25%).

In relation to pathological variables, 52% of our population had stage III-IV, and 72% had tumor grade III at diagnosis. These values are higher compared to other studies in different geographic scenarios. In India, Sarin *et al*. [18] reported that 30.1% of TNBC cases had stage III-IV at diagnosis, while in China, Li *et al*. [13] reported that 35% of their population had histologic grade III at diagnosis. In the United States, a population-based study found that 22% of TNBC patients presented with stage III-IV, and 75% had histologic grade III at diagnosis [19]. One explanation for the higher proportion of tumor stage in our cohort may be related to the unwillingness of the women to participate in breast cancer screening programs and engage in regular cancer-preventive medical checks, which has shown leads to cancer propagation and increased tumor severity at diagnosis [20]. A previous study in Peruvian females found that poverty is correlated with higher tumor stage at diagnosis [21], which supports our assumption. One previous study in a Peruvian population found similar results, with 42% being in stage III-IV and 70% with histologic grade III, demonstrating a trend to these outcomes in this population [3]. Moreover, 32% of the population did not undergo surgery. This is because half of our patients were diagnosed at advanced stages or progressed during the neoadjuvant chemotherapy. Hence, although there are recommendations of case by case evaluation for the surgical approach, if the disease is at stage IV or do not respond to chemotherapy, the main objective would be improving the quality of life and the survival benefit of tumor removal is still inconclusive [22, 23].

Patients with TNBC have a lower DFS and OS than those with non-TNBC [4, 24]. Previous studies have reported a DFS of 70–80% and an OS of 70–80% at five years [4, 10, 13, 25–27]. However, few authors have focused on long-term survival outcomes at seven [13, 28], eight [11], and 20 years [12]. Similar to our median eight-year follow-up, Li *et al*. [13] reported 62% of DFS at nine years in a cohort of 129 Chinese patients with TNBC. Moreover, Chandra *et al*. [11] found a significant difference between OS rates of early (90%) and locally advanced breast cancer (51%) at eight-years, despite a median follow-up of 42 months. Furthermore, Haque *et al*. [12] reported an OS of 86% in patients with tumor stage I and a 63% OS in stage II at 20 years, which is similar to the patients in this cohort (stage I, 85%; and stage II, 75%). In a similar cohort of 255 Latin American patients with TNBC, Vallejos *et al*. [3] reported an OS of 66% at five years; however, we analyzed a wider population and expanded the observation period.

Cox regression identified that tumor size, nodal status, and chemotherapy and radiotherapy are important prognostic factors for both disease recurrence and mortality. Similarly, Abdulkarim *et al*. [29] analyzed 768 TNBC patients and reported that at least three positive lymph nodes increased the risk of local recurrence by 9-fold and the risk of mortality by 7-fold. In a cohort study of 605 cases, Leon-Ferre *et al*. [25] identified that a higher N status increases the risk of invasive recurrence, while Bhatti *et al*. [10] reported that both tumor size and a higher N status increased the risk of local-distant relapse and mortality in a cohort of 194 women with TNBC. The worse DFS and OS in more advanced cancer stages are congruent with the heterogeneous and aggressive behavior of TNBC [12, 30]. Our results remain consistent with these previous experiences and portray the prognostic factors for EFS in Peruvian population.

In the present study, we found that radiotherapy improved the OS of the patients. Previous studies established the benefits of this treatment in survival outcomes such as locoregional recurrence and among types of surgery [29, 31]. In our analysis radiotherapy was an additional prognostic factor to reduce the risk of mortality in the TNBC population. Another favorable prognostic factor for EFS and OS was treatment with chemotherapy. Despite the lack of targeted therapy, according to international guidelines chemotherapy was essential for the treatment of these patients and proved to be a favorable prognostic marker for this type of tumor [32, 33]. However even though new chemotherapy agents such as platines, microtubule stabilizers and monoclonal antibodies have been added to this treatment, TNBC patients still have the worst prognosis across the different phenotypes of breast cancer [34, 35].

A family history of breast or ovarian cancer is a confirmed prognostic factor for breast cancer, and it has been described that patients with TNBC have a higher proportion of family members with these diagnoses [7, 36]. However, few studies have addressed the importance of this factor in the prognosis of breast cancer [37, 38]. *Song et al*. [37] performed a meta-analysis in which they aimed to evaluate the influence of breast cancer history in a first-degree relative and the risk of OS and breast cancer-specific survival. They identified that a positive family history is a protective factor for OS. The authors stated that possible implications in their analysis were the quality of the studies and the heterogeneous control groups studied. In contrast, we only analyzed TNBC patients and included the outcome EFS. Although a positive family history of breast or ovarian cancer in a first- or second-degree relative tended to be a protective factor in both EFS and OS outcomes, there was also a tendency towards a worse prognosis. We focused on the triple-negative subtype in contrast to the study of Song *et al*., therefore, the difference in the expression of hormonal receptors in the patients may explain these different results. Moreover, the percentage of positive family histories in our population was smaller than that of other studies [12, 13, 38, 39], which could have affected the results. Due to the underreported data of survival outcomes, a stratified analysis based on hormonal receptors would provide better understanding of the implication of this variable in the survival of breast cancer patients.

The present study has several strengths. Previous experiences used different definitions for both OS and EFS. While some authors defined it as the time from diagnosis until the event (death or recurrence) [12, 13, 26, 40], others start the follow-up from the treatment date (medical or surgery) [10, 29, 41]. This latter approach prevents incurring an immortal time bias in the survival analysis by avoiding the observation time of patients without therapy. In this line, we tracked the follow-up of our patients from the date of diagnosis, hence, our results also demonstrate the long-term effectiveness of the breast cancer treatment modalities in this population. Moreover, our study included a large number of Peruvian cases, providing the sociodemographic and clinicopathological profiles of these patients. On the other hand, one limitation of our study is its retrospective design. The clinical and treatment characteristics in the medical records of many patients were incomplete or were lost during follow-up and these patients were, therefore, excluded from the analysis. While there was a high proportion of patients with positive margins in the BCS group, all received re-excision and medical treatment, which could have affected the survival outcomes in this cohort. Moreover, this was a single center experience and our results should be interpreted with caution. Our results are useful for the Peruvian or South American people, but they have to be considered with caution if extrapolated to other populations.

In conclusion, the clinicodemographic and pathological features of our TNBC patients are similar to those of other populations. However, most of our patients were diagnosed in stage III, which likely explains the lower EFS and OS rates observed. The prognostic factors of survival in the present study were also similar to those described in previous studies. The most important prognostic factor identified was the lymph node stage. New specific molecular biomarkers able to predict response to chemotherapy are needed for new therapies in patients with TNBC.

## Supporting information

S1 Table Sociodemographic, pathological, treatment and outcomes of triple negative breast cancer patients according to neoadjuvant chemotherapy. (DOCX)

S2 Table Univariate and multivariate analysis for overall survival from patients who received neoadjuvant chemotherapy. (DOCX)

S1 Data (SAV)

## References

[1] Bray F, Ferlay J, Soerjomataram I, Siegel RL, Torre LA, Jemal A. Global cancer statistics 2018: GLOBOCAN estimates of incidence and mortality worldwide for 36 cancers in 185 countries. CA Cancer J Clin. 2018;68(6):394–424. 10.3322/caac.21492 30207593

[2] National Cancer Institute—Surveillance, Epidemiology, and End Results Program,. Cancer Stat Facts: Female Breast Cancer [Internet]. NIH; [cited 2019 October 27]. Available from: https://seer.cancer.gov/statfacts/html/breast.html.

[3] Vallejos CS, Gomez HL, Cruz WR, Pinto JA, Dyer RR, Velarde R, et al Breast cancer classification according to immunohistochemistry markers: subtypes and association with clinicopathologic variables in a peruvian hospital database. Clinical breast cancer. 2010;10(4):294–300. 10.3816/CBC.2010.n.038 . 20705562

[4] Onitilo AA, Engel JM, Greenlee RT, Mukesh BN. Breast cancer subtypes based on ER/PR and Her2 expression: comparison of clinicopathologic features and survival. Clinical medicine & research. 2009;7(1–2):4–13. 10.3121/cmr.2009.825 19574486 PMC2705275

[5] Bauer KR, Brown M, Cress RD, Parise CA, Caggiano V. Descriptive analysis of estrogen receptor (ER)-negative, progesterone receptor (PR)-negative, and HER2-negative invasive breast cancer, the so-called triple-negative phenotype: a population-based study from the California cancer Registry. Cancer. 2007;109(9):1721–8. 10.1002/cncr.22618 . 17387718

[6] Trivers KF, Lund MJ, Porter PL, Liff JM, Flagg EW, Coates RJ, et al The epidemiology of triple-negative breast cancer, including race. Cancer causes & control: CCC. 2009;20(7):1071–82. 10.1007/s10552-009-9331-1 19343511 PMC4852686

[7] Anderson K, Thompson PA, Wertheim BC, Martin L, Komenaka IK, Bondy M, et al Family history of breast and ovarian cancer and triple negative subtype in hispanic/latina women. SpringerPlus. 2014;3:727 Epub 2015/02/26. 10.1186/2193-1801-3-727 25713754 PMC4332916

[8] Tamayo LI, Vidaurre T, Navarro Vásquez J, Casavilca S, Aramburu Palomino JI, Calderon M, et al Breast cancer subtype and survival among Indigenous American women in Peru. PLoS One. 2018;13(9):e0201287–e. 10.1371/journal.pone.0201287 . 30183706 PMC6124707

[9] Marker KM, Zavala VA, Vidaurre T, Lott PC, Vásquez JN, Casavilca-Zambrano S, et al Human Epidermal Growth Factor Receptor 2–Positive Breast Cancer Is Associated with Indigenous American Ancestry in Latin American Women. Cancer Research. 2020 10.1158/0008-5472.CAN-19-3659 32245796 PMC7202960

[10] Bhatti AB, Khan AI, Siddiqui N, Muzaffar N, Syed AA, Shah MA, et al Outcomes of triple-negative versus non-triple-negative breast cancers managed with breast-conserving therapy. Asian Pacific journal of cancer prevention: APJCP. 2014;15(6):2577–81. 10.7314/apjcp.2014.15.6.2577 . 24761867

[11] Chandra D, Suresh P, Sinha R, Azam S, Batra U, Talwar V, et al Eight Year Survival Analysis of Patients with Triple Negative Breast Cancer in India. Asian Pacific journal of cancer prevention: APJCP. 2016;17(6):2995–9. Epub 2016/07/01. doi: APJCP.2016.17.6.2995 . 27356724

[12] Haque R, Ahmed SA, Inzhakova G, Shi J, Avila C, Polikoff J, et al Impact of breast cancer subtypes and treatment on survival: an analysis spanning two decades. Cancer epidemiology, biomarkers & prevention: a publication of the American Association for Cancer Research, cosponsored by the American Society of Preventive Oncology. 2012;21(10):1848–55. Epub 2012/09/20. 10.1158/1055-9965.epi-12-0474 22989461 PMC3467337

[13] Li CY, Zhang S, Zhang XB, Wang P, Hou GF, Zhang J. Clinicopathological and prognostic characteristics of triple- negative breast cancer (TNBC) in Chinese patients: a retrospective study. Asian Pacific journal of cancer prevention: APJCP. 2013;14(6):3779–84. 10.7314/apjcp.2013.14.6.3779 . 23886182

[14] Salazar MR, Regalado-Rafael R, Navarro JM, Montanez DM, Abugattas JE, Vidaurre T. [The role of the National Institute of Neoplastic Diseases in the control of cancer in Peru]. Rev Peru Med Exp Salud Publica. 2013;30(1):105–12. Epub 2013/04/25. 10.1590/s1726-46342013000100020 . 23612822

[15] Singh JC, Lichtman SM. Effect of age on drug metabolism in women with breast cancer. Expert Opin Drug Metab Toxicol. 2015;11(5):757–66. 10.1517/17425255.2015.1037277 . 25940027 PMC5057182

[16] Marosi C, Köller M. Challenge of cancer in the elderly. ESMO Open. 2016;1(3):e000020 10.1136/esmoopen-2015-000020 27843603 PMC5070391

[17] Mowad R, Chu QD, Li BD, Burton GV, Ampil FL, Kim RH. Does obesity have an effect on outcomes in triple-negative breast cancer? The Journal of surgical research. 2013;184(1):253–9. Epub 2013/06/19. 10.1016/j.jss.2013.05.037 . 23768767

[18] Sarin R, Khandrika L, Hanitha R, Avula A, Batra M, Kaul S, et al Epidemiological and survival analysis of triple-negative breast cancer cases in a retrospective multicenter study. Indian journal of cancer. 2016;53(3):353–9. 10.4103/0019-509X.200682 . 28244455

[19] Li X, Yang J, Peng L, Sahin AA, Huo L, Ward KC, et al Triple-negative breast cancer has worse overall survival and cause-specific survival than non-triple-negative breast cancer. Breast cancer research and treatment. 2017;161(2):279–87. 10.1007/s10549-016-4059-6 . 27888421

[20] Kolak A, Kaminska M, Sygit K, Budny A, Surdyka D, Kukielka-Budny B, et al Primary and secondary prevention of breast cancer. Annals of agricultural and environmental medicine: AAEM. 2017;24(4):549–53. Epub 2017/12/30. 10.26444/aaem/75943 . 29284222

[21] Gutiérrez C, Alarcón E. Nivel de pobreza asociado al estadio de gravedad del cáncer ginecológico. Anales de la Facultad de Medicina. 2008;69:239–43.

[22] Somsekhar SP, Geeta K, Jain R, Nayyer R, Halder S, Malik VK, et al Practical consensus recommendations regarding role of mastectomy in metastatic breast cancer. South Asian J Cancer. 2018;7(2):79–82. 10.4103/sajc.sajc_106_18 . 29721468 PMC5909300

[23] Rashid OM, Takabe K. Does removal of the primary tumor in metastatic breast cancer improve survival? J Womens Health (Larchmt). 2014;23(2):184–8. Epub 2013/11/21. 10.1089/jwh.2013.4517 . 24261650 PMC3922396

[24] Pal S, Luchtenborg M, Davies EA, Jack RH. The treatment and survival of patients with triple negative breast cancer in a London population. SpringerPlus. 2014;3:553 Epub 2014/10/18. 10.1186/2193-1801-3-553 25324980 PMC4188837

[25] Leon-Ferre RA, Polley MY, Liu H, Gilbert JA, Cafourek V, Hillman DW, et al Impact of histopathology, tumor-infiltrating lymphocytes, and adjuvant chemotherapy on prognosis of triple-negative breast cancer. Breast cancer research and treatment. 2018;167(1):89–99. 10.1007/s10549-017-4499-7 28913760 PMC5790598

[26] Yadav S, Ladkany R, Yadav D, Alhalabi O, Khaddam S, Isaac D, et al Impact of BRCA Mutation Status on Survival of Women With Triple-negative Breast Cancer. Clinical breast cancer. 2018;18(5):e1229–e35. 10.1016/j.clbc.2017.12.014 . 29402697

[27] Kaplan HG, Malmgren JA, Atwood MK. Triple-negative breast cancer in the elderly: Prognosis and treatment. The breast journal. 2017;23(6):630–7. 10.1111/tbj.12813 . 28485826

[28] Joyce DP, Murphy D, Lowery AJ, Curran C, Barry K, Malone C, et al Prospective comparison of outcome after treatment for triple-negative and non-triple-negative breast cancer. The surgeon: journal of the Royal Colleges of Surgeons of Edinburgh and Ireland. 2017;15(5):272–7. 10.1016/j.surge.2016.10.001 . 28277293

[29] Abdulkarim BS, Cuartero J, Hanson J, Deschenes J, Lesniak D, Sabri S. Increased risk of locoregional recurrence for women with T1-2N0 triple-negative breast cancer treated with modified radical mastectomy without adjuvant radiation therapy compared with breast-conserving therapy. Journal of clinical oncology: official journal of the American Society of Clinical Oncology. 2011;29(21):2852–8. 10.1200/JCO.2010.33.4714 21670451 PMC5073381

[30] Dolle JM, Daling JR, White E, Brinton LA, Doody DR, Porter PL, et al Risk factors for triple-negative breast cancer in women under the age of 45 years. Cancer epidemiology, biomarkers & prevention: a publication of the American Association for Cancer Research, cosponsored by the American Society of Preventive Oncology. 2009;18(4):1157–66. 10.1158/1055-9965.EPI-08-1005 19336554 PMC2754710

[31] O'Rorke MA, Murray LJ, Brand JS, Bhoo-Pathy N. The value of adjuvant radiotherapy on survival and recurrence in triple-negative breast cancer: A systematic review and meta-analysis of 5507 patients. Cancer treatment reviews. 2016;47:12–21. 10.1016/j.ctrv.2016.05.001 . 27214603

[32] Ismail-Khan R, Bui MM. A review of triple-negative breast cancer. Cancer Control. 2010;17(3):173–6. Epub 2010/07/29. 10.1177/107327481001700305 . 20664514

[33] Li Y, Yang D, Chen P, Yin X, Sun J, Li H, et al Efficacy and safety of neoadjuvant chemotherapy regimens for triple-negative breast cancer: a network meta-analysis. Aging. 2019;11(16):6286–311. Epub 2019/08/24. 10.18632/aging.102188 . 31446432 PMC6738404

[34] Mehanna J, Haddad FG, Eid R, Lambertini M, Kourie HR. Triple-negative breast cancer: current perspective on the evolving therapeutic landscape. Int J Womens Health. 2019;11:431–7. 10.2147/IJWH.S178349 . 31447592 PMC6682754

[35] Lebert JM, Lester R, Powell E, Seal M, McCarthy J. Advances in the systemic treatment of triple-negative breast cancer. Curr Oncol. 2018;25(Suppl 1):S142–S50. Epub 2018/06/13. 10.3747/co.25.3954 . 29910657 PMC6001760

[36] Bethea TN, Rosenberg L, Castro-Webb N, Lunetta KL, Sucheston-Campbell LE, Ruiz-Narvaez EA, et al Family History of Cancer in Relation to Breast Cancer Subtypes in African American Women. Cancer epidemiology, biomarkers & prevention: a publication of the American Association for Cancer Research, cosponsored by the American Society of Preventive Oncology. 2016;25(2):366–73. 10.1158/1055-9965.EPI-15-1068 26721669 PMC4767636

[37] Song JL, Chen C, Yuan JP, Sun SR. The association between prognosis of breast cancer and first-degree family history of breast or ovarian cancer: a systematic review and meta-analysis. Familial cancer. 2017;16(3):339–49. 10.1007/s10689-017-9969-x . 28176206

[38] Phipps AI, Buist DS, Malone KE, Barlow WE, Porter PL, Kerlikowske K, et al Family history of breast cancer in first-degree relatives and triple-negative breast cancer risk. Breast cancer research and treatment. 2011;126(3):671–8. 10.1007/s10549-010-1148-9 20814817 PMC3059326

[39] Asleh-Aburaya K, Fried G. Clinical and molecular characteristics of triple-negative breast cancer patients in Northern Israel: single center experience. SpringerPlus. 2015;4:132 10.1186/s40064-015-0900-3 25825688 PMC4372619

[40] Rayson D, Payne JI, Michael JCR, Tsuruda KM, Abdolell M, Barnes PJ. Impact of Detection Method and Age on Survival Outcomes in Triple-Negative Breast Cancer: A Population-Based Cohort Analysis. Clinical breast cancer. 2018;18(5):e955–e60. 10.1016/j.clbc.2018.04.013 . 29885790

[41] Khalifa J, Duprez-Paumier R, Filleron T, Lacroix Triki M, Jouve E, Dalenc F, et al Outcome of pN0 Triple-Negative Breast Cancer with or without Lymph Node Irradiation: A Single Institution Experience. The breast journal. 2016;22(5):510–9. 10.1111/tbj.12626 . 27261365

