# Supplementary material for: Triple-negative breast cancer in Peru: 2000 patients and 15 years of experience
Source: PLoS One. 2020 Aug 24;15(8):e0237811. doi: 10.1371/journal.pone.0237811 (PMC7444821; doi:10.1371/journal.pone.0237811)
Supplement: S1 Table — (DOCX) [file pone.0237811.s001.docx]

**S1 Table.** Sociodemographic, pathological, treatment and outcomes of triple negative breast cancer patients according to neoadjuvant chemotherapy.

| Characteristics | Neoadjuvant Chemotherapy | | P-value |
| --- | --- | --- | --- |
|  | No  N (%)  N=1263 | Yes  N (%)  N=744 |  |
| Age (years) |  |  | **0.022** |
| ≤35 | 145 (11.5) | 98 (13.2) |  |
| 36-49 | 479 (37.9) | 317 (42.6) |  |
| ≥50 | 639 (50.6) | 329 (44.2) |  |
| Menopausal status |  |  |  |
| Premenopause | 527 (42.2) | 362 (49.3) | **0.002** |
| Postmenopause | 722 (57.8) | 373 (50.7) |  |
| Family history of breast and/or ovarian cancer |  |  |  |
| No | 1089 (86.2) | 652 (87.6) | 0.37 |
| Yes | 174 (13.8) | 92 (12.4) |  |
| T staging |  |  |  |
| T1-2 | 761 (66.1) | 104 (14.0) | **<0.0001** |
| T3-4 | 390 (33.9) | 637 (86.0) |  |
| N staging |  |  | **<0.0001** |
| N0 | 515 (45.1) | 136 (18.7) |  |
| N1 | 343 (30.1) | 372 (51.2) |  |
| N2 | 155 (13.6) | 147 (20.2) |  |
| N3 | 128 (11.2) | 72 (9.9) |  |
| Tumor stage |  |  | **<0.0001** |
| Stage I | 138 (11.1) | 6 (0.8) |  |
| Stage II | 597 (48.0) | 90 (12.1) |  |
| Stage III | 423 (34.0) | 601 (81.0) |  |
| Stage IV | 76 (6.9) | 45 (6.1) |  |
| Histologic grade |  |  | **0.003** |
| Grade I-II | 231 (21.1) | 92 (15.2) |  |
| Grade III | 863 (78.9) | 515 (84.8) |  |
| Radiotherapy |  |  | **<0.0001** |
| No | 689 (54.6) | 306 (41.1) |  |
| Yes | 574 (45.5) | 438 (58.9) |  |
| Loco-regional recurrence |  |  | **<0.0001** |
| No | 901 (71.3) | 429 (57.7) |  |
| Yes | 362 (28.7) | 315 (42.3) |  |
| Distant recurrence |  |  | **<0.0001** |
| No | 745 (59.0) | 215 (28.9) |  |
| Yes | 518 (41.0) | 529 (71.1) |  |
| Death |  |  | **<0.0001** |
| No | 620 (49.1) | 187 (25.1) |  |
| Yes | 643 (50.9) | 557 (74.9) |  |
