# Supplementary material for: Triple-negative breast cancer in Peru: 2000 patients and 15 years of experience
Source: PLoS One. 2020 Aug 24;15(8):e0237811. doi: 10.1371/journal.pone.0237811 (PMC7444821; doi:10.1371/journal.pone.0237811)
Supplement: S2 Table — (DOCX) [file pone.0237811.s002.docx]

**S2 Table.** Univariate and multivariate analysis for overall survival from patients who received neoadjuvant chemotherapy.

| **Characteristics** | **Overall Survival** | | | | | |
| --- | --- | --- | --- | --- | --- | --- |
|  | **Analysis (c)** | | | **Analysis (a)** | | |
|  | **cHR** | **95% CI** | **p value** | **aHR** | **95% CI** | **p value** |
| Age | 1.00 | 0.99-1.01 | 0.59 | 1.00 | 0.99-1.01 | 0.94 |
| Family history of breast and/or ovarian cancer |  |  |  |  |  |  |
| No | 1.00 |  |  | 1.00 |  |  |
| Yes | 0.90 | 0.70-1.17 | 0.43 | 0.97 | 0.72-1.31 | 0.85 |
| T staging |  |  |  |  |  |  |
| T1-2 | 1.00 |  |  | 1.00 |  |  |
| T3-4 | 2.00 | 1.52-2.63 | **<0.001** | 1.77 | 1.29-2.44 | **<0.001** |
| N staging |  |  |  |  |  |  |
| N0 | 1.00 |  |  | 1.00 |  |  |
| N1 | 1.55 | 1.21-1.98 | **<0.001** | 1.30 | 0.99-1.69 | 0.054 |
| N2 | 2.04 | 1.54-2.70 | **<0.001** | 1.68 | 1.24-2.29 | **<0.001** |
| N3 | 3.02 | 2.18-4.19 | **<0.001** | 2.70 | 1.87-3.88 | **<0.001** |
| Histologic grade |  |  |  |  |  |  |
| Grade I-II | 1.00 |  |  | 1.00 |  |  |
| Grade III | 0.87 | 0.68-1.12 | 0.28 | 0.85 | 0.66-1.10 | 0.21 |
| Radiotherapy |  |  |  |  |  |  |
| No | 1.00 |  |  | 1.00 |  |  |
| Yes | 0.65 | 0.55-0.76 | **<0.001** | 0.69 | 0.57-0.84 | **<0.001** |
